# Editorial: The physiological response of aquatic invertebrates to pollution

**DOI:** 10.3389/fphys.2023.1295636

**Published:** 2023-09-26

**Authors:** M. G. Lionetto, V. Matozzo

**Affiliations:** ^1^ Department Biological and Environmental Sciences and Technologies (DiSTeBA), Salento University, Lecce, Italy; ^2^ National Biodiversity Future Center (NBFC), Palermo, Italy; ^3^ Department of Biology, School of Sciences, University of Padua, Padua, Italy

**Keywords:** invertebrate, pollution, physiological response, microplastics, metals, pesticide, effluent

Chemical pollution, derived from either point and non-point pollution sources, represents one of the main threats to aquatic environments. Chemical pollutants such as trace metals, oil-based products, pesticides, fertilizers, antifouling compounds, plastic materials, pharmaceutical and veterinary products represent a worldwide menace to the health of aquatic organisms (Tornero and Hanke, 2016).

Many physiological responses are developed by the organisms at the molecular, cellular, and organism levels to cope with environmental pollution, including activation of detoxification processes, repair responses, metabolic changes, and alterations of physiological functions. The information gained through these responses can facilitate our understanding of the mechanistic basis of pollutant toxicity, organisms’ compensation, and acclimatization processes, and may be useful in predicting pollutant impact on the biota. Therefore, the detection of the physiological responses to pollutants and their quantification represents an important and useful tool to assess the quality of the environment and to support biodiversity conservation strategies. Among aquatic organisms, invertebrates represent most of the species, with 97% of the species living in marine habitats and 3% in fresh-water habitats (Rosner et al., 2023). They perform crucial ecological functions in all aquatic ecosystems (Palumbi et al., 2009) having colonized a large spectrum of ecological niches. This information highlights the importance of the study of invertebrate response to pollution in aquatic environments for assessing the impact of environmental stressors on biodiversity and natural resources. The advances in “omics” and bioinformatics methodologies in the last decade provide powerful new approaches for fast and effectively acquiring important knowledge about the mechanistic mode of action of pollutants, single or in a mixture, and their impact on the organisms’ physiological status (Lionetto et al., 2021).

The Research Topic entitled “*The physiological response of aquatic invertebrates to pollution*” published in Frontiers in Physiology provides emerging knowledge into this multifaceted research field.

Among aquatic pollutants, microplastics/nanoplastics have become a priority issue in recent years due to their widespread distribution into the environment and raise concerns about their potential toxicity to aquatic organisms. Little is known in the literature on the long-term effects of micro-nano plastics on aquatic organisms and on possible acclimation mechanisms to counteract the effects of microplastic/nanoplastic exposure. Rades et al. focused on this field by studying the long-term responses of reef-building stony corals to microplastics. In these organisms, it is known that exposure to microplastics is associated with negative health effects (Reichert et al., 2021) including growth reduction (Hankins et al., 2018), feeding rate lowering (Corinaldesi et al., 2021), alteration in the immune system (Tang et al., 2018). On a long-term scale, the authors did not find any acclimatization process in the feeding and defense behavior that could help corals to better cope with this environmental stressor. Therefore, they suggest that, in the absence of any acclimation process, microplastic pollution might constitute a constant stressor for coral organisms, likely leading to sustained energy costs and health impairment. The work of Almulhim et al. opens perspectives for a deeper understanding of the physiology of aquatic organisms through a metabolomic approach. The author deepened the analysis of the *Tridacna maxima* giant clam, a significant component of tropical Indo-Pacific coral reef communities (Neo et al., 2015), and illustrates the power of invertebrate metabolite profiling for monitoring plastic-related aquatic pollutants.

Moreover, the Research Topic focuses on coastal and estuarine environments that are heavily influenced by natural and anthropogenic activities and greatly affected by chemical pollution. In particular, pesticides used in agriculture and aquaculture and chemicals used as biocides in antifouling paints are the most diffuse coastal pollutants, representing a serious threat to the physiology of non-target organisms. Daoud et al. analyzed the effects on the development, growth, and metabolism of lobster at stage IV (*Homarus americanus*) following chronic exposure to sediments spiked with commercial formulations of deltamethrin and permethrin, common pyrethroid pesticides used on crops as well as applied to aquaculture pens. Both compounds caused an increased frequency of malformations. While permethrin’s main effects were sublethal, deltamethrin increased lobster mortality. The results highlight the potential of pesticides released into the coastal areas for effects on non-target marine organisms. The paper of Cima and Varello focuses on antifouling compounds, in particular copper-based antifouling paints whose worldwide diffusion has increased copper release into coastal environments and its potential impact on either target or non-target organisms. This work analyzes the immunotoxic effects of the exposure to the antifouling copper(I) biocide on target and non-target bivalve species with a comparative *in vitro* approach on 1h cultured hemocytes of *Mytilus galloprovincialis,* a dominant species in the macrofouling community, and *Ruditapes philippinarum*, a non-target species of antifouling paint chemicals. The authors suggest a potential mechanism of oxidative stress-immunotoxicity-cell toxicity of Cu(I) ions in these two species, involving both direct and indirect interactions with copper ions. The analysis of the cellular and molecular responses developed by the hemocytes of the two species towards antifouling copper(I) biocide highlights the higher sensitivity of the non-target species, raising great concern for the potentially negative effects of antifouling biocides on the survival of non-target key species in the coastal environment.

The work of André et al. takes into consideration another aspect of great concern for aquatic environment pollution, such as the impact on the biota of municipal effluents which are well-recognized for their interfering activity on sexual differentiation and reproduction (André et al., 2020). In particular, the authors examined the combined effects of sewer overflow, which is increased by rain due to climate change, and municipal effluents on freshwater *Elliptio complanata* mussels.

In conclusion, the study of “*the physiological response of aquatic invertebrates to pollution*” is a multifaceted emerging research field whose insight can give a valuable contribution to the understanding of the impact, resistance, and resilience of the organisms to chemical pollution pressure also in consideration of the global climate changes.
